# Left Bundle Branch Area Pacing in Cardiac Resynchronization Therapy: How Does It Compare to Biventricular Pacing in Terms of Electrocardiographic Parameters and Procedural Outcomes?

**DOI:** 10.3390/jcm15010200

**Published:** 2025-12-26

**Authors:** Tariel Atabekov, Sergey Krivolapov, Roman Batalov, Sergey Popov

**Affiliations:** Cardiology Research Institute, Tomsk National Research Medical Center, Russian Academy of Sciences, Kievskaya St., 111a, 634012 Tomsk, Russiapsv@cardio-tomsk.ru (S.P.)

**Keywords:** cardiac resynchronization therapy, biventricular pacing, left bundle branch area pacing, electrocardiographic and procedural outcomes

## Abstract

**Background/Objectives**: Biventricular pacing (BVP) to deliver cardiac resynchronization therapy (CRT) is a standard intervention for heart failure, yet suboptimal response remains common due to challenges in left ventricular (LV) lead placement. Left bundle branch area pacing (LBBAP) has emerged as a promising alternative, offering physiological activation via direct conduction system engagement. However, comparative data on electrocardiographic (ECG) and procedural outcomes between LBBAP-CRT and BVP-CRT are limited. **Methods**: This retrospective, single-center study compared LBBAP-CRT and BVP-CRT in 114 patients with left bundle branch block and LV ejection fraction ≤ 35%. LBBAP-CRT was performed using a Medtronic SelectSecure™ 3830 lead via a fixed-curve sheath Medtronic C315HIS, with successful capture confirmed by ECG criteria (Qr/qR in V1, LV activation time < 100 ms). BVP-CRT involved coronary sinus LV lead placement. Outcomes included QRS duration, pacing thresholds, complications, and procedural metrics. Statistical analysis employed logistic regression to identify predictors of optimal pacing thresholds (≤1.0 V at 0.5 ms). **Results**: LBBAP-CRT yielded greater degree of QRS narrowing than BVP-CRT (136.7 ± 13.5 ms vs. 147.2 ± 14.6 ms, *p* < 0.001) and lower pacing thresholds (*p* < 0.05). Complications occurred in 18.1% of BVP-CRT patients (phrenic nerve stimulation, lead dislocation) versus none in LBBAP-CRT (*p* = 0.011). According to the multivariable analysis LBBAP-CRT was associated with an optimal thresholds (*p* = 0.007), alongside lower E/e′ ratio and lead impedance. **Conclusions**: LBBAP-CRT was associated with superior electrical resynchronization, fewer complications, and better pacing thresholds compared to BVP, suggesting its potential as a preferred CRT strategy. Larger randomized trials are needed to validate long-term outcomes.

## 1. Introduction

Cardiac resynchronization therapy (CRT) is a standard treatment for patients with heart failure (HF) and electrical dyssynchrony, typically delivered via biventricular pacing (BVP) [1]. Despite its proven benefits, a significant proportion of patients exhibit suboptimal response, often due to challenges in achieving optimal left ventricular (LV) lead placement and effective resynchronization [2]. In recent years, left bundle branch area pacing (LBBAP) has emerged as a promising alternative to conventional BVP, offering a more physiological approach to ventricular activation by directly engaging the conduction system [3].

LBBAP has demonstrated favorable outcomes in terms of lead stability, pacing thresholds, and correction of QRS duration in patients requiring CRT [4]. However, comparative data on electrocardiographic (ECG) parameters and procedural outcomes between LBBAP-CRT and BVP-CRT remain limited. While BVP-CRT relies on simultaneous stimulation of the right ventricle (RV) and LV to restore synchrony, LBBAP-CRT aims to restore intrinsic conduction by capturing the left bundle branch, potentially leading to more physiological ventricular activation [5].

This study seeks to compare LBBAP-CRT and BVP-CRT by evaluating key ECG parameters (such as QRS duration, morphology, and electrical resynchronization) as well as procedural outcomes (including lead success rates, complications, and pacing thresholds). Understanding these differences may help refine patient selection and optimize CRT delivery, ultimately improving clinical outcomes.

## 2. Materials and Methods

### 2.1. Study Design and Population

This retrospective, observational, single-center study compared the real-world clinical outcomes of BVP-CRT and LBBAP-CRT in HF patients. The study was performed at the Cardiology Research Institute, Russia between December 2021 and May 2025, the study included patients who successfully underwent CRT via BVP or LBBAP.

Eligible participants had a sinus rhythm, symptomatic heart failure (New York Heart Association [NYHA] class II and III), a left ventricular ejection fraction (LVEF) ≤ 35%, and met Strauss criteria for left bundle branch block (LBBB), with an indication for CRT-defibrillator (CRT-D) implantation. Exclusion criteria comprised age < 18 years, prior CRT-D implantation, or unsuccessful CRT-D placement.

### 2.2. Consent

All patients provided written informed consent after being informed that LBBAP-CRT represents a nonstandard approach. The choice between BVP-CRT and LBBAP-CRT was based on operator preference.

All study procedures complied with the ethical principles outlined in the Declaration of Helsinki and adhered to Good Clinical Practice guidelines. The study protocol received formal approval from the Local Ethics Committee of the Cardiology Research Institute (Protocol No. 219, 26 October 2021, and Protocol No. 240, 15 February 2023). Most participants were enrolled from registered clinical trials (ClinicalTrials.gov identifiers: NCT03667989 and NCT05769036). All participants provided written informed consent for the publication of their clinical data.

### 2.3. Implantation Technique and Device Programming for LBBAP-CRT

The defibrillation lead, an active-fixation model, was implanted first and positioned at either the right ventricular septum or apex. Following this, the atrial lead was placed, secured at the appendage or the upper third of the right atrium. Lastly, the lead for LBBAP-CRT was implanted. The placement of all leads was carried out by the implanting physician following standard procedural guidelines, using fluoroscopic guidance and a transvenous approach. We assessed lead performance by measuring capture thresholds and sensing amplitudes with a pacing system analyzer (Medtronic, Minneapolis, MN, USA) connected via sterile crocodile-clip cables.

LBBAP-CRT was performed using the SelectSecure™ pacing lead (Model 3830, 69 cm, Medtronic, Mounds View, MN, USA) delivered via a fixed-curve sheath (C315 HIS, Medtronic, Mounds View, MN, USA), as previously described [6]. The lead was initially positioned on the RV side of the interventricular septum (IVS) and then advanced deep into the septum until reaching the LV subendocardium. Successful LBBAP was confirmed by the following criteria: QRS morphology in ECG lead V1 showing a Qr or qR pattern, pacing impedance ≥ 300 Ohm, and stimulus-to-LV activation time (LVAT) in leads V5/V6 < 100 ms at low pacing output (≤3 V/0.5 ms) [6,7,8,9,10]. The lead was repositioned if septal perforation was suspected, which was indicated by pacing impedance values fewer than 300 Ohms or a sudden loss of capture.

Continuous monitoring included 12-lead ECG, intracardiac electrograms, and fluoroscopy. For CRT-D implantation, the LBBAP lead was connected to the LV port, while the defibrillator and atrial leads were placed in the RV/DF and right atrial (RA) ports, respectively. Post-implant, devices were programmed in DDD mode, with LV tip to RV coil/Can pace configuration, and with atrioventricular (AV) delay optimized to achieve the narrowest QRS duration, ensuring exclusive LBBAP capture. CRT-D programming followed established international guidelines [11].

### 2.4. Implantation Technique and Device Programming for BVP-CRT

The defibrillation and atrial leads were positioned using the previously outlined implantation technique. To visualize the branch of cardiac veins, retrograde balloon occluded venograms of the coronary sinus were routinely obtained. The preferred sites for passive-fixation LV lead placement were the lateral, posterolateral, or anterolateral branches, following standard clinical protocols. In all patients in BVP-CRT group a quadripolar LV lead was used. The RV pacing/defibrillation lead, RA lead, and coronary sinus LV lead were connected to their respective ports on the device.

Devices were initially set to DDD mode to maintain an appropriate biventricular pacing percentage, with optimization of the AV delay. The optimal LV pacing vector was selected based on threshold testing, avoidance of phrenic nerve stimulation, and assessment of paced QRS duration. After implantation, devices remained in DDD mode, with AV and interventricular (VV) delays adjusted to achieve the narrowest possible QRS complex.

### 2.5. Study Procedures and Device Follow-Up Protocol

Patient baseline characteristics, including demographic data, medical background, current pharmacotherapy, ECG findings, and echocardiographic parameters were collected. Procedure duration and fluoroscopy time were defined from initial C315 HIS sheath insertion to successful LBBAP lead placement, and from sheath advancement to completion of coronary sinus LV lead implantation in the BVP-CRT group, respectively.

Post-implantation device evaluation was performed the day after implantation for all participants, assessing pacing thresholds (volts [V]), sensing amplitudes (millivolts [mV]), and lead impedances (Ohms). For coronary sinus LV/LBBAP leads, a pacing threshold ≤ 1.0 V at 0.5 ms was considered optimal [2,5]. All pacing threshold measurements and subsequent amplitude programming were performed using a fixed pulse width of 0.5 ms. Adverse events related to lead placement were monitored. Standard 12-lead ECGs were obtained during implantation and post-procedure, with QRS duration and LVAT measurements measured from pacing stimulus onset to QRS termination and V5/V6 R-wave peak, respectively, at 25 mm/s paper speed.

### 2.6. Statistical Analysis and Risk Stratification

Data are summarized as counts and percentages for categorical variables and as mean (M) ± standard deviation (SD) or median (Me) [interquartile range (Q1; Q3)] for continuous variables, depending on data distribution. Descriptive analyses were performed for the overall cohort and separately for the LBBAP-CRT and BVP-CRT groups. Intergroup comparisons were conducted using chi-square or Fisher’s exact tests for categorical variables and independent *t*-tests or Mann–Whitney U tests for continuous variables, as appropriate. For paired comparisons of dependent samples, we employed the Wilcoxon signed-rank test.

To identify predictors of optimal pacing threshold (defined as ≤1.0 V at 0.5 ms pulse width), we performed a two-stage regression analysis: univariable screening and multivariable modeling. First, all clinically relevant independent variables were initially assessed using univariable logistic regression. Variables demonstrating significant association (*p* < 0.05) with the primary outcome were selected for further analysis. Candidate variables from the univariable analysis were entered into a stepwise multivariable logistic regression model. We assessed multicollinearity using variance inflation factors (<5 considered acceptable). Model fit was evaluated using the Hosmer–Lemeshow test (*p* > 0.05 indicating adequate fit). To exclude potential confounders, we tested the independent variables for collinearity and examined significant correlations between predictors and other measurements using Spearman analysis. Results from our regression analysis are presented as odds ratios (OR) with 95% confidence intervals (CI). To evaluate the discriminatory power of optimal pacing threshold-associated variables, we computed the area under the curve (AUC).

All analyses were performed using MedCalc (version 19.2.6, MedCalc Software, Ostend, Belgium) and Statistica (version 10.0, StatSoft Inc., Tulsa, OK, USA). A *p*-value of <0.05 was considered to be significant.

## 3. Results

### 3.1. Patient Enrollment and Group Allocation

**Figure 1:**
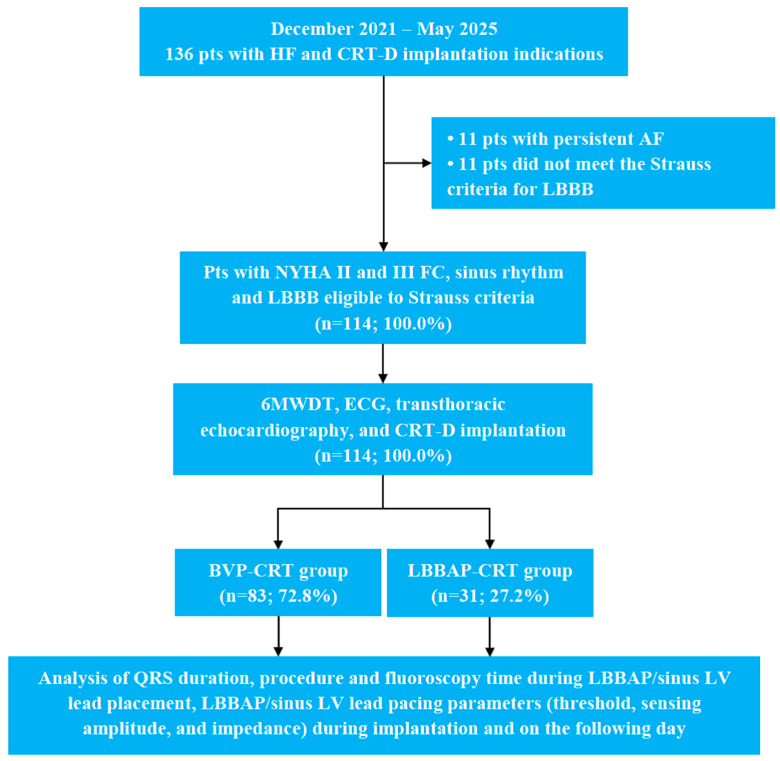
The study design and flowchart. 6MWDT, 6 min walk distance test; AF, atrial fibrillation; BVP, biventricular pacing; CRT, cardiac resynchronization therapy; CRT-D, cardiac resynchronization therapy devices with the defibrillation function; ECG, electrocardiography; FC, functional class; HF, heart failure; LBBAP, left bundle branch area pacing; LBBB, left bundle branch block; LV, left ventricular; NYHA, New York Heart Association.

A flowchart depicting the study design and participant selection process is provided in Figure 1. Among 136 initially screened patients, 22 were excluded (11 due to persistent atrial fibrillation and 11 who did not meet the Strauss criteria for LBBB). The remaining 114 patients were included in the study and divided into two groups: 83 patients received BVP-CRT, while 31 underwent LBBAP-CRT.

Comprehensive baseline characteristics of the study population are summarized in Table 1. The cohort had a mean age of 61.4 ± 11.0 years with predominantly male participants (81.1%). Ischemic HF was present in 31.6% of cases, nonischemic in 49.1%, while 19.3% exhibited mixed etiology. Dyslipidemia was documented in 51.8% of patients overall, with significantly higher prevalence in the BVP-CRT group compared to LBBAP-CRT (59.0% vs. 32.2%, *p* = 0.011). All participants received guideline-directed medical therapy, with comparable treatment profiles between groups except for angiotensin II receptor blockers, which were more frequently prescribed in the BVP-CRT group (18.1% vs. 0%, *p* = 0.011). Baseline echocardiographic and electrocardiographic parameters showed mean LVEF of 28.7 ± 4.9% and QRS duration of 180.5 ± 25.4 ms for the entire cohort. Notably, LVEF was significantly lower in BVP-CRT recipients compared to LBBAP-CRT patients (28.2 ± 5.0% vs. 30.3 ± 4.5%, *p* = 0.042). No other statistically significant intergroup differences in baseline parameters were identified.

### 3.2. Procedural Outcomes and Early Post-Implantation Device Assessment

**Figure 2:**
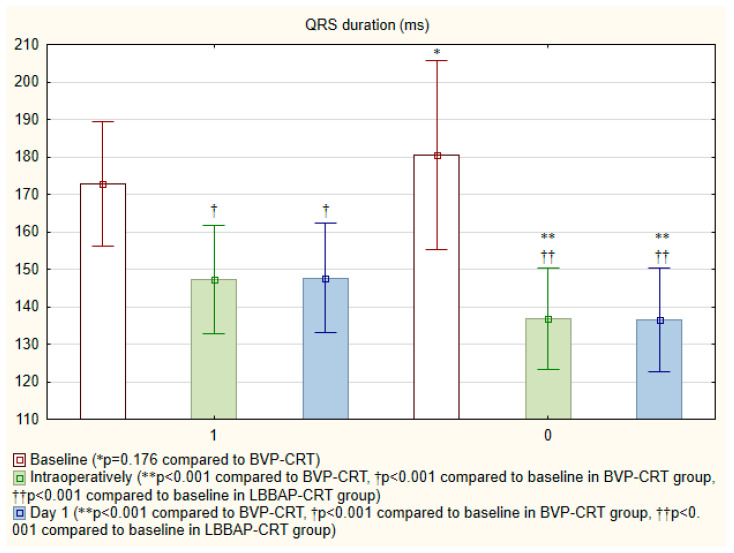
Comparison of QRS duration changes following BVP-CRT (1) and LBBAP-CRT (0) implantation. BVP, biventricular pacing; CRT, cardiac resynchronization therapy; LBBAP, left bundle branch area pacing.

**Figure 3:**
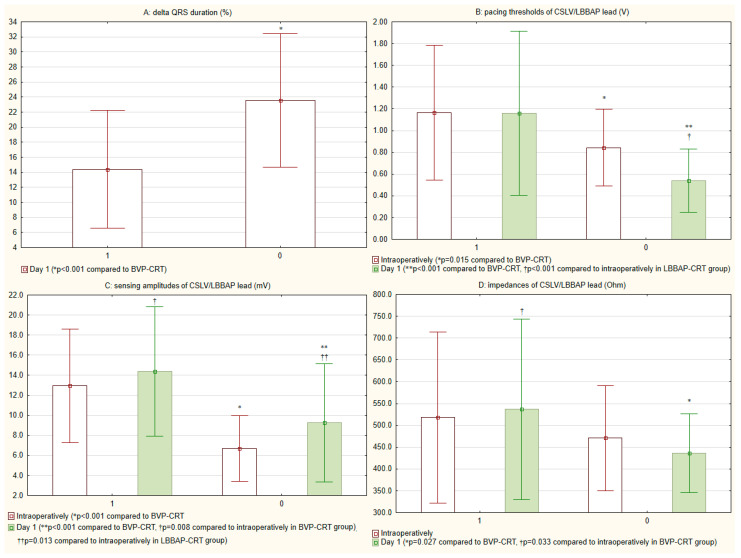
Comparison of (**A**) QRS duration delta and changes in lead (**B**) pacing thresholds, (**C**) sensing amplitudes and (**D**) impedances: BVP-CRT (1) vs. LBBAP-CRT (0). BVP, biventricular pacing; CRT, cardiac resynchronization therapy; CSLV, coronary sinus left ventricular; LBBAP, left bundle branch area pacing.

Among all 114 patients receiving CRT-D, BVP-CRT was used in 72.8% (n = 83) and LBBAP-CRT in 27.2% (n = 31). No patients crossed over from BVP-CRT to LBBAP-CRT or vice versa. Coronary sinus LV lead implantation duration was longer in the BVP-CRT group than LBBAP lead placement in the LBBAP-CRT group (23.8 [20.0; 27.0] vs. 22.0 [16.0; 25.8] minutes, respectively; *p* = 0.167), though this difference was not statistically significant. Fluoroscopy times were similar for coronary sinus LV lead implantation and LBBAP lead placement (5.0 [3.3; 6.6] vs. 5.0 [4.0; 8.7] minutes, *p* = 0.642). Mean LVAT in the LBBAP-CRT group was 68.9 ± 12.6 ms. The LBBAP-CRT was associated with significantly shorter paced QRS duration than BVP-CRT (Figure 2 and Figure 3A), both intraoperatively (136.7 ± 13.5 ms vs. 147.2 ± 14.6 ms) and the day after implantation (136.5 ± 13.7 ms vs. 147.6 ± 14.6 ms; *p* < 0.001 for both comparisons).

LBBAP-CRT was associated with significantly lower capture thresholds (*p* < 0.05) and sensing amplitudes (*p* < 0.05) compared to coronary sinus LV leads at implantation and the day after implantation (Figure 3B,C). While impedance values were similar initially (*p* = 0.491), LBBAP leads showed significantly lower impedances postoperatively (*p* = 0.027) (Figure 3D).

No acute procedural complications were recorded in the LBBAP-CRT group (0%), compared to an 18.1% complication rate in the BVP-CRT group (*p* = 0.011). Specific complications in the BVP-CRT group included coronary vein dissection (2.4%), phrenic nerve stimulation (14.4%), and coronary sinus LV lead dislocation (1.2%); all occurred more frequently than with LBBAP-CRT (all *p* > 0.05 except phrenic stimulation, *p* = 0.026). One patient (1.2%) required lead revision for coronary sinus lead dislocation in the BVP-CRT group versus none in the LBBAP-CRT group (*p* = 0.541). No lead perforations occurred in the LBBAP-CRT group postoperatively. Detailed procedural characteristics are shown in Table 2.

### 3.3. Risk Stratification by Optimal Pacing Threshold Analysis

**Figure 4:**
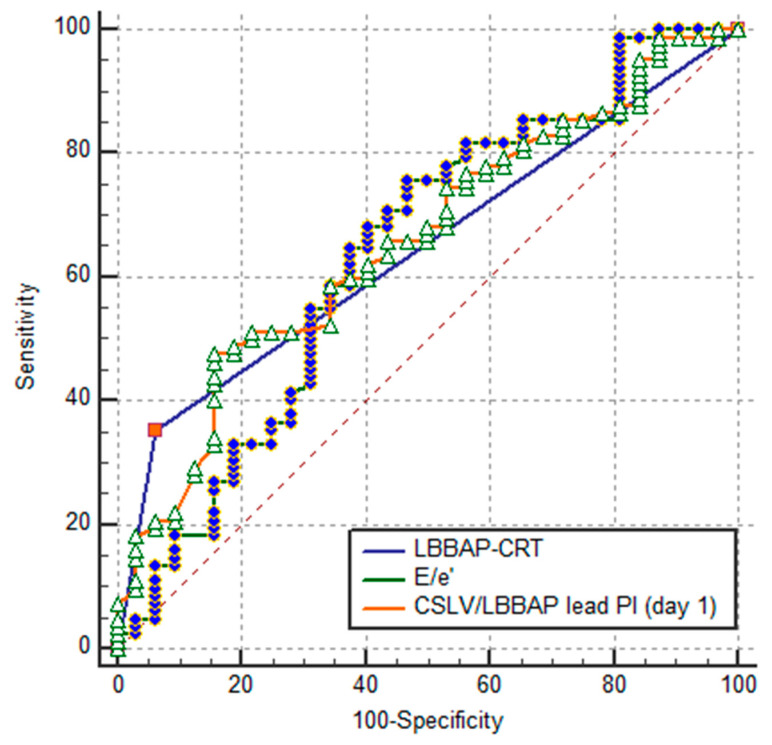
Comparison of ROC curves for assessing the ability of variables to distinguish between CRT patients with CSLV/LBBAP lead pacing threshold 1 V or less and those with more than 1 V. CRT, cardiac resynchronization therapy; E/e′, left ventricle early diastolic filling velocity to early diastolic motion velocity of the mitral annulus ratio; LBBAP, left bundle branch area pacing; PI, pacing impedance.

Among the analyzed variables, LBBAP-CRT was associated with achieving an optimal lead pacing threshold. Receiver operating characteristic (ROC) analysis revealed that LBBAP-CRT could discriminate cases with optimal thresholds with an AUC of 0.646 (95% CI: 0.550–0.733; Figure 4). Other parameters showed comparable discriminative performance: E/e′ ratio (AUC = 0.639 [95% CI: 0.544–0.727]), coronary sinus LV/LBBAP lead pacing impedance (postoperative day 1) (AUC = 0.656 [95% CI: 0.562–0.743]), delta QRS duration (postoperative day 1) (AUC = 0.637 [95% CI: 0.542–0.725]), angiotensin receptor neprilysin inhibitor (ARNI) use (AUC = 0.612 [95% CI: 0.516–0.702]), and loop diuretics use (AUC = 0.601 [95% CI: 0.505–0.691]).

**Figure 5:**
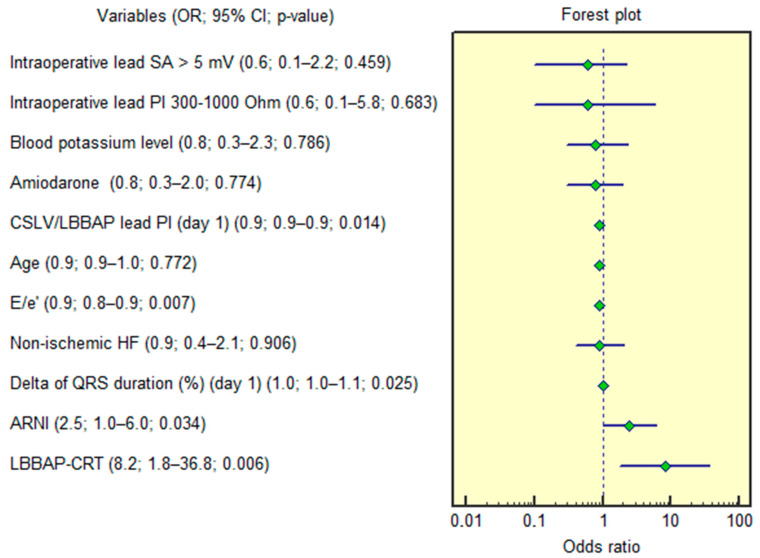
Forest plot illustrating the results of the univariable logistic regression analysis. 95% CI, 95% confidence interval; ARNI, angiotensin receptor neprilysin inhibitor; CRT, cardiac resynchronization therapy; CSLV, coronary sinus left ventricular; E/e′, left ventricle early diastolic filling velocity to early diastolic motion velocity of the mitral annulus ratio; HF, heart failure; LBBAP, left bundle branch area pacing; OR, odds ratio; PI, pacing impedance; SA, sensing amplitude.

Univariable analysis (Figure 5) identified several factors associated with optimal lead pacing thresholds: LBBAP-CRT (OR = 8.2; 95% CI 1.8–36.8; *p* = 0.006), E/e′ ratio (OR = 0.9; 95% CI 0.8–0.9; *p* = 0.007), CSLV/LBBAP lead impedance (postoperative day 1) (OR = 0.9; 95% CI 0.9–0.9; *p* = 0.014); ΔQRS duration (postoperative day 1) (OR = 1.0; 95% CI 1.0–1.1; *p* = 0.025), ARNI use (OR = 2.5; 95% CI 1.0–6.0; *p* = 0.034). In multivariable analysis (adjusted for non-ischemic HF, serum potassium, amiodarone use, age, intraoperative sensing amplitude > 5 mV, and pacing impedance 300–1000 Ω), only three variables retained significance: LBBAP-CRT (OR = 9.7; 95% CI 1.8–50.9; *p* = 0.007), E/e′ ratio (OR = 0.8; 95% CI 0.8–0.9; *p* = 0.002), CSLV/LBBAP lead impedance (OR = 0.9; 95% CI 0.9–0.9; *p* = 0.036).

## 4. Discussion

The present study provides a comprehensive comparison between patients with LBBAP-CRT and conventional BVP-CRT. Our findings highlight several key differences in ECG parameters, procedural outcomes, and pacing lead performance, supporting LBBAP-CRT as a viable and potentially superior alternative to BVP-CRT in select patients.

A major finding of our study was the more pronounced reduction in QRS duration in the LBBAP-CRT group compared to BVP-CRT, both intraoperatively and on postoperative day 1 (136.7 ± 13.5 ms vs. 147.2 ± 14.6 ms, *p* < 0.001). This observation aligns with the foundational principle of Left bundle branch area pacing optimized CRT (LOT-CRT), as conceptualized by Leventopoulos et al. [12]. LOT-CRT represents an advanced strategy beyond simple LBBAP-CRT, intentionally leveraging the fusion of intrinsic right bundle conduction with paced left bundle stimulation to create a truly “wavefront-based” resynchronization. Our narrower QRS complexes likely reflect successful engagement of this optimized fusion, moving beyond mere correction of LBBB towards a more physiological, heart-rate-adaptive activation sequence. This is a critical distinction from BVP-CRT, which creates an obligatory, non-physiological electrical wavefront irrespective of intrinsic conduction. In LBBB-CRT patients, Mamedova et al. found that isolated left ventricular pacing which narrowed the QRS complex also reduced mechanical dyssynchrony and enhanced both global and segmental left ventricular contractility [13]. Our observation results aligns with prior studies demonstrating that LBBAP-CRT results in more physiological ventricular activation by directly engaging the His-Purkinje system, thereby improving electrical resynchronization [7,8,14]. The narrower QRS complex in LBBAP-CRT patients suggests more efficient depolarization, which may translate into better mechanical synchrony and improved clinical outcomes [15].

Additionally, the LVAT in the LBBAP-CRT group (68.9 ± 12.6 ms) was consistent with prior reports of successful conduction system capture [5]. The shorter LVAT further supports the notion that LBBAP-CRT achieves rapid and synchronous LV activation, which is often difficult to attain with traditional BVP-CRT due to variable coronary sinus lead placement and delayed LV stimulation [16].

Our study demonstrated notable differences in procedural success and complication rates between the two pacing strategies. While fluoroscopy times and lead placement durations were comparable, LBBAP-CRT was associated with a significantly lower complication rate (0% vs. 18.1%, *p* = 0.011). Specifically, BVP-CRT was more frequently complicated by phrenic nerve stimulation (14.4% vs. 0%, *p* = 0.026), coronary vein dissection (2.4%), and lead dislocations (1.2%). These findings are consistent with previous reports highlighting the technical challenges of coronary sinus lead placement, including anatomical variations and lead instability [17,18,19].

LBBAP-CRT was associated with superior pacing thresholds compared to coronary sinus LV leads (*p* < 0.05), with a higher likelihood of achieving optimal thresholds (≤1.0 V at 0.5 ms). This advantage persisted postoperatively, reinforcing the stability and efficiency of LBBAP leads. The lower impedance observed in LBBAP leads (*p* = 0.027) may reflect differences in lead-tissue interface characteristics, though further studies are needed to explore long-term implications.

According to the multivariable regression analysis LBBAP-CRT was associated with achieving an optimal pacing threshold (OR = 9.7, 95% CI: 1.8–50.9, *p* = 0.007). Other significant predictors included lower E/e′ ratio (indicative of better diastolic function) and lower lead impedance. These findings suggest that LBBAP-CRT not only offers better acute electrical performance but may also be influenced by underlying cardiac structural and functional status.

However, it is crucial to contextualize our findings within an important limitation of the current evidence base, as highlighted by Vlachakis et al. [20]. The vast majority of studies, including ours, have focused exclusively on patients with HF and reduced LVEF (HFrEF). This focus, while clinically imperative for the traditional CRT population, may overlook a broader potential paradigm shift. Vlachakis et al. [20] argue that conduction system pacing, including LBBAP-CRT/LOT-CRT, may have significant implications for patients with HF with preserved LVEF (HFpEF), particularly those with LBBB and evidence of mechanical dyssynchrony or diastolic impairment. Our multivariable analysis, which identified a lower E/e′ ratio (indicative of better diastolic function) as a predictor of optimal pacing performance, indirectly supports this notion. It suggests that the benefits of physiological activation may extend beyond systolic augmentation to include improved diastolic synchrony. Therefore, the current evidence, while robust for HFrEF, may represent only the first chapter in understanding the full therapeutic scope of LBBAP-CRT and LOT-CRT.

While our study did not record device infections as a specific endpoint and the sample size and follow-up duration were not powered to detect differences in this low-frequency event, the procedural implications of our findings have theoretical relevance for infection risk. Device infections are among the most serious complications of cardiac implantable electronic device therapy, with procedural complexity, operative duration, number of leads, and need for re-intervention being established risk factors [21]. In our cohort, the LBBAP-CRT strategy achieved comparable electrical resynchronization, shorter procedural times, and a lower overall acute complication rate requiring re-intervention (e.g., no coronary sinus dissections or lead dislodgements). This streamlined approach, avoiding coronary sinus manipulation, may translate into a reduced risk of periprocedural contamination and subsequent infection. This hypothesis is supported by strategies aimed at reducing infection burden, such as the use of antibiotic-impregnated collagen sponges [22]. While prospective, long-term data are needed to confirm this potential ancillary benefit, the simplified procedural architecture of LBBAP-CRT presents a compelling theoretical advantage in mitigating a major source of morbidity, mortality, and cost in device therapy.

The growing body of evidence, including our study, supports LBBAP-CRT as a promising alternative to BVP-CRT. The physiological activation pattern, lower complication rates, and superior lead performance make LBBAP-CRT particularly appealing for patients with challenging coronary sinus anatomy or those at high risk for BVP-CRT related complications. However, broader adoption of LBBAP requires further validation in larger randomized trials to assess long-term clinical outcomes, including heart failure hospitalization rates and mortality [23,24,25].

Our findings contribute to the evolving paradigm in CRT. LBBAP-CRT offers more physiological resynchronization, a favorable safety profile, and excellent lead performance, affirming its role as a superior alternative to BVP-CRT for patients with HFrEF and LBBB. Future efforts must focus on three key areas: (1) conducting large-scale randomized trials to solidify its long-term superiority in HFrEF; (2) standardizing implantation protocols and definitions for LOT-CRT, as emphasized by Leventopoulos et al. [12]; and (3) expanding research, as proposed by Vlachakis et al. [20], to explore its efficacy in broader patient phenotypes, including those with HFpEF and conduction disorders, to truly assess the scope of this paradigm shift.

### 4.1. Novelty of the Study, Clinical Implications and Future Perspectives

While many studies have described LBBAP-CRT, our study provides a head-to-head, single-center comparison with conventional BVP-CRT in a real-world HF cohort implanted during the same period, minimizing the impact of evolving general cardiology care.

Beyond simple QRS duration, we provided a detailed analysis of electrocardiographic parameters including the rates of true QRS morphology transformation to right bundle branch block pattern (suggesting direct LBB area capture), QRS fragmentation, and paced morphology consistency as potential markers of lead placement quality and electrical resynchronization.

We reported detailed “real-world” procedural times and success rates, contributing to the growing literature on the learning curve and practical implementation of LBBAP-CRT versus a well-established BVP-CRT approach.

Our study, by its design, inadvertently highlights the current real-world dilemma of modality choice based on operator preference, setting the stage for the discussion on the need for randomized trials and better selection algorithms.

### 4.2. Study Limitations

This study has several important limitations that should inform the interpretation of its results. First, its retrospective, single-center, non-randomized design introduces the potential for significant selection bias. The choice between LBBAP-CRT and BVP-CRT was based on operator preference, which may have systematically influenced patient allocation. For instance, operators may have preferentially selected LBBAP-CRT for patients with anticipated challenges in coronary sinus anatomy or prior failed BVP-CRT attempts, while opting for BVP-CRT for more anatomically straightforward cases. If true, the comparable or superior procedural success of LBBAP-CRT becomes even more notable, as it was achieved in a potentially more complex cohort. Conversely, this selection bias limits the direct comparability of the groups and may confound the interpretation of electrical outcomes like QRS narrowing, which could be influenced by differences in underlying cardiac substrate.

Second, despite statistical adjustments, residual confounding likely persists. Observed baseline differences between groups (e.g., lower LVEF in the BVP-CRT cohort) may reflect underlying variations in cardiomyopathy etiology, fibrosis burden, or cardiovascular risk profiles that independently influence both procedural outcomes and clinical response. Our analysis cannot fully account for these nuanced clinical factors or for unmeasured confounders related to operator decision-making.

Third, the safety profile of LBBAP-CRT requires cautious interpretation. The absence of recorded complications in the LBBAP-CRT group is encouraging but must be viewed in the context of the cohort’s relatively small sample size (n = 31), which limits the precision of this safety estimate. Furthermore, if operator selection reserved LBBAP-CRT for anatomically favorable candidates, the observed low complication rate may not be fully generalizable. Definitive conclusions regarding comparative safety await validation from larger, randomized studies.

Finally, technical expertise and generalizability are key considerations. LBBAP-CRT is a technically demanding procedure with a recognized learning curve, requiring proficiency in septal lead deployment and electrophysiological confirmation of left bundle branch capture. The favorable outcomes reported here stem from a center with dedicated experience and may not be immediately replicable in early-adoption settings. Broader implementation will necessitate standardized protocols and specific training.

Consequently, while our findings contribute supportive evidence for LBBAP-CRT, they underscore the imperative for larger, prospective, randomized controlled trials with long-term follow-up to definitively establish its comparative efficacy, safety, and durability.

## 5. Conclusions

In this real-world comparison, LBBAP-CRT was associated with superior electrical resynchronization, lower complication rates, and better pacing thresholds compared to conventional BVP-CRT in HF patients. These findings suggest that LBBAP-CRT may be a preferable strategy in appropriately selected patients. Future randomized controlled trials are warranted to confirm these benefits and refine patient selection criteria.

## Figures and Tables

**Table 1:** Baseline demographic and clinical characteristics of the overall study population and stratified groups.

Characteristics	Overall Population (n = 114)	BVP-CRT Group (n = 83)	LBBAP-CRT Group (n = 31)	*p*_2–3_
	1	2	3	
Age, year, M ± SD	61.4 ± 11.0	59.8 ± 11.2	60.7 ± 8.4	0.386
Male gender, n (%)	83 (72.8)	55 (66.3)	28 (90.3)	0.066
Ischemic heart failure, n (%)	36 (31.6)	25 (30.1)	11 (35.5)	0.585
Non-ischemic heart failure, n (%)	56 (49.1)	45 (54.2)	11 (35.5)	0.076
Mixed etiology of heart failure, n (%)	22 (19.3)	13 (15.7)	9 (29.0)	0.109
Coronary artery stenting in anamnesis, n (%)	32 (28.1)	22 (26.5)	10 (32.2)	0.545
6 min walk distance test, m, M ± SD	296.7 ± 66.5	295.5 ± 64.5	299.9 ± 72.3	0.709
Systolic blood pressure, mmHg, M ± SD	120.0 ± 9.0	120.1 ± 6.0	119.6 ± 14.4	0.944
Diastolic blood pressure, mmHg, M ± SD	74.7 ± 6.2	74.8 ± 4.7	74.4 ± 9.3	0.816
Body mass index, kg/m^2^, M ± SD	28.3 ± 5.2	28.4 ± 5.2	27.8 ± 5.2	0.424
Estimated GFR, mL/min/1.73 m^2^, M ± SD	72.5 ± 19.2	72.0 ± 18.4	74.0 ± 21.5	0.539
New York Heart Association functional class of heart failure:				
II, n (%)	56 (49.1)	40 (48.2)	16 (51.6)	0.746
III, n (%)	58 (50.9)	43 (51.8)	15 (48.4)	0.746
QRS duration, ms, M ± SD	174.8 ± 19.5	172.7 ± 16.6	180.5 ± 25.4	0.176
LVESV, ml, M ± SD	170.3 ± 54.8	174.4 ± 52.5	159.2 ± 59.8	0.111
Left ventricular ejection fraction, %, M ± SD	28.7 ± 4.9	28.2 ± 5.0	30.3 ± 4.5	0.042
E/e′, Me [Q1; Q3]	11.9 [8.8; 17.6]	12.0 [8.2; 17.6]	11.6 [9.1; 18.8]	0.923
Pre-implantation arrhythmias:				
Paroxysmal atrial fibrillation, n (%)	34 (29.8)	27 (32.5)	7 (22.6)	0.304
Sustained ventricular tachycardia, n (%)	9 (7.9)	5 (6.0)	4 (12.9)	0.227
Ventricular fibrillation, n (%)	4 (3.5)	3 (3.6)	1 (3.2)	0.920
Paroxysmal SVT, n (%)	3 (2.6)	2 (2.4)	1 (3.2)	0.809
Comorbidities:				
Diabetes mellitus, n (%)	24 (21.1)	15 (18.1)	9 (29.0)	0.203
Dyslipidemia, n (%)	59 (51.8)	49 (59.0)	10 (32.2)	0.011
Stroke, n (%)	5 (4.4)	4 (4.8)	1 (3.2)	0.713
Smoking, n (%)	39 (34.2)	25 (30.1)	14 (45.2)	0.133
Therapy:				
Beta-blockers, n (%)	108 (94.7)	80 (96.4)	28 (90.3)	0.199
Loop diuretics, n (%)	69 (60.5)	50 (60.2)	19 (61.3)	0.922
Mineralocorticoid receptor antagonists, n (%)	108 (94.7)	78 (94.0)	30 (96.8)	0.559
ACEIs, n (%)	42 (36.8)	31 (37.3)	11 (35.5)	0.855
Antiplatelet agents, n (%)	65 (57.0)	47 (56.6)	18 (58.1)	0.891
Lipid-lowering treatment, n (%)	93 (81.6)	70 (84.3)	23 (74.2)	0.216
ARNI, n (%)	54 (47.4)	36 (43.4)	18 (58.1)	0.164
Angiotensin II receptor blockers, n (%)	15 (13.2)	15 (18.1)	0 (0.0)	0.011
Amiodarone, n (%)	51 (44.7)	39 (47.0)	12 (38.7)	0.431
Anticoagulants, n (%)	44 (38.6)	33 (39.7)	11 (35.5)	0.678
Ivabradine, n (%)	4 (3.5)	4 (4.8)	0 (0.0)	0.215
SGLT2Is, n (%)	102 (89.5)	72 (86.7)	30 (96.8)	0.122

Values are expressed as M ± SD for continuous variables and n (%) for categorical variables. Abbreviations: ACEIs, angiotensin-converting enzyme inhibitors; ARNI, angiotensin receptor neprilysin inhibitor; BVP, biventricular pacing; CRT, cardiac resynchronization therapy; E/e′, left ventricle early diastolic filling velocity to early diastolic motion velocity of the mitral annulus ratio; GFR, glomerular filtration rate; LBBAP, left bundle branch area pacing; LVESV, left ventricular end-systolic volume; SGLT2Is, sodium glucose co-transporter 2 inhibitors; SVT, supraventricular tachycardia.

**Table 2:** The procedural data for the overall cohort and by treatment group (BVP-CRT vs. LBBAP-CRT).

Data	Overall Cohort (n = 114)	BVP-CRT Group (n = 83)	LBBAP-CRT Group (n = 31)	*p*_2–3_
	1	2	3	
CSLV/LBBAP lead implantation duration, min, Me [Q1; Q3]	23.0 [19.0; 26.4]	23.8 [20.0; 27.0]	22.0 [16.0; 25.8]	0.167
Fluoroscopy time for CSLV/LBBAP lead implantation, min, Me [Q1; Q3]	5.0 [3.3; 6.7]	5.0 [3.3; 6.6]	5.0 [4.0; 8.7]	0.642
Pacing threshold of CSLV/LBBAP lead:				
Implant, V, M ± SD	1.08 ± 0.58	1.16 ± 0.62	0.84 ± 0.35	0.015
First day after implantation, V, M ± SD	0.99 ± 0.72	1.16 ± 0.76	0.54 ± 0.30	<0.001
Sensing amplitude of CSLV/LBBAP lead:				
Implant, mV, M ± SD	11.2 ± 5.8	12.9 ± 5.6	6.7 ± 3.3	<0.001
First day after implantation, mV, M ± SD	13.0 ± 6.7	14.4 ± 6.4	9.2 ± 5.9	<0.001
Impedance of CSLV/LBBAP lead:				
Implant, Ohm, M ± SD	505.2 ± 179.7	518.1 ± 196.4	470.6 ± 120.5	0.491
First day after implantation, Ohm, M ± SD	509.6 ± 187.8	537.1 ± 207.0	436.2 ± 90.3	0.027
LVAT, ms, M ± SD	-	-	68.9 ± 12.6	-
Paced QRS duration:				
Implant, ms, M ± SD	144.4 ± 15.0	147.2 ± 14.6	136.7 ± 13.5	<0.001
First day after implantation, ms, M ± SD	144.6 ± 15.2	147.6 ± 14.6	136.5 ± 13.7	<0.001
Delta QRS duration:				
Implant, %, M ± SD	16.9 ± 9.1	14.4 ± 7.8	23.5 ± 8.9	<0.001
First day after implantation, %, M ± SD	16.9 ± 9.1	14.4 ± 7.8	23.6 ± 8.9	<0.001
Procedural complications:				
Total, n (%)	15 (13.2)	15 (18.1)	0 (0.0)	0.011
Coronary vein dissection, n (%)	2 (1.8)	2 (2.4)	0 (0.0)	0.385
Phrenic nerve stimulation, n (%)	12 (10.5)	12 (14.4)	0 (0.0)	0.026
CSLV/LBBAP lead dislocation, n (%)	1 (0.9)	1 (2.4)	0 (0.0)	0.541

Values are expressed as M ± SD or Me [Q1; Q3] for continuous variables and n (%) for categorical variables. Abbreviations: CRT, cardiac resynchronization therapy; CSLV, coronary sinus left ventricular; LBBAP, left bundle branch area pacing.

## Data Availability

The datasets generated and/or analyzed during this study are available from the corresponding author upon reasonable request.

## Abbreviations

The following abbreviations are used in this manuscript:

ARNI	Angiotensin Receptor Neprilysin Inhibitor
AUC	Area Under the ROC Curve
AV	Atrioventricular
BVP	Biventricular Pacing
CI	Confidence Interval
CRT	Cardiac Resynchronization Therapy
CRT-D	CRT-defibrillator
ECG	Electrocardiography
HF	Heart Failure
IVS	Interventricular Septum
LBBAP	Left Bundle Branch Area Pacing
LBBB	Left Bundle Branch Block
LV	Left Ventricular
LVAT	Left Ventricular Activation Time
LVEF	Left Ventricular Ejection Fraction
M	Mean
Me	Median
NYHA	New York Heart Association
OR	Odds ratio
RA	Right Atrial
ROC	Receiver Operating Characteristic
RV	Right Ventricle
SD	Standard Deviation
